# A Drop of Danger: A Subtle Sign of an Entrapped Pulmonary Artery Catheter

**DOI:** 10.7759/cureus.101455

**Published:** 2026-01-13

**Authors:** Harish Ram

**Affiliations:** 1 Anesthesiology, University of Kentucky, Lexington, USA

**Keywords:** bleeding catheter, bleeding pulmonary artery catheter, entrapped catheter, entrapped pulmonary artery catheter, pulmonary artery catheter complication

## Abstract

Awareness of potential complications associated with a pulmonary artery catheter (PAC) is essential to minimize the risk of adverse outcomes. Entrapment of a PAC is an uncommon but serious event, most frequently reported during cardiac surgery. However, it is usually recognized postoperatively when resistance is encountered during catheter repositioning or removal. Once identified, management often necessitates re-operation, although percutaneous techniques may be used in some cases. This report presents an atypical case of PAC entrapment, emphasizing the importance of early detection and outlining strategies for prevention in the intraoperative period.

## Introduction

The pulmonary artery catheter (PAC) is currently employed as a selective monitoring tool in patients with marked hemodynamic instability and in those who are critically ill [1-3]. Its use is indicated when advanced hemodynamic assessment is expected to guide clinical decisions regarding fluid management, inotropic support, vasopressor therapy, or pulmonary vasodilator administration based on PAC-derived measurements. However, PAC use carries inherent risks, ranging from common but generally benign electrical disturbances to rare yet potentially life-threatening vascular complications. The overall incidence of serious complications in surgical patients is estimated to be between 0.1% and 0.5% [4].

PAC entrapment is an uncommon but serious complication, most frequently reported in the context of cardiac surgery. With a reported incidence of approximately <0.068%, which is likely underestimated due to underreporting, it typically occurs intraoperatively but is often recognized during the postoperative period [5]. This report details a unique instance of intraoperatively detected entrapment that presented unusually and highlights the importance of being vigilant of subtle clues of PAC complications.

## Case presentation

A 41-year-old woman with severe mitral regurgitation and moderate mitral stenosis secondary to rheumatic heart disease, and superimposed infective endocarditis, presented for elective mitral valve replacement (MVR). Past medical history was significant for hepatitis C and left tibial osteomyelitis. Following application of standard American Society of Anesthesiologists monitors [6] and placement of a right radial arterial catheter, general anesthesia was induced without complication. A transesophageal echocardiography (TEE) probe was inserted, after which a right internal jugular two-lumen central venous catheter with an introducer sheath and a 7.5 F continuous cardiac output monitoring PAC were placed uneventfully. Placement was guided by pressure waveform analysis, with TEE employed when difficulty was encountered or when the first placement attempt was unsuccessful.

**Figure 1:**
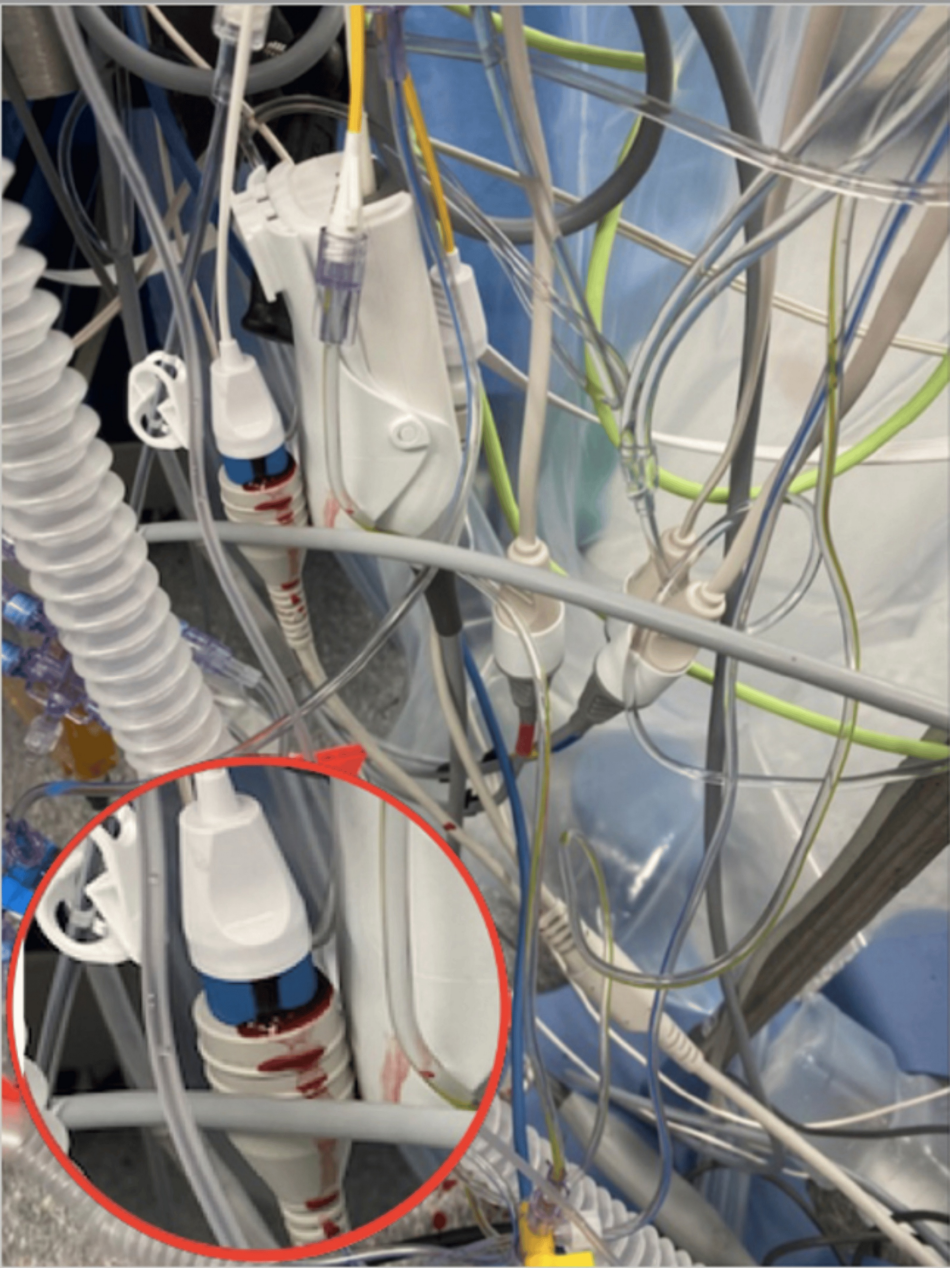
Blood at the connector port The presence of blood was noted at the PAC thermistor connector port. (The insert in the bottom left is a zoomed-in view of the connector port). PAC: pulmonary artery catheter

**Figure 2:**
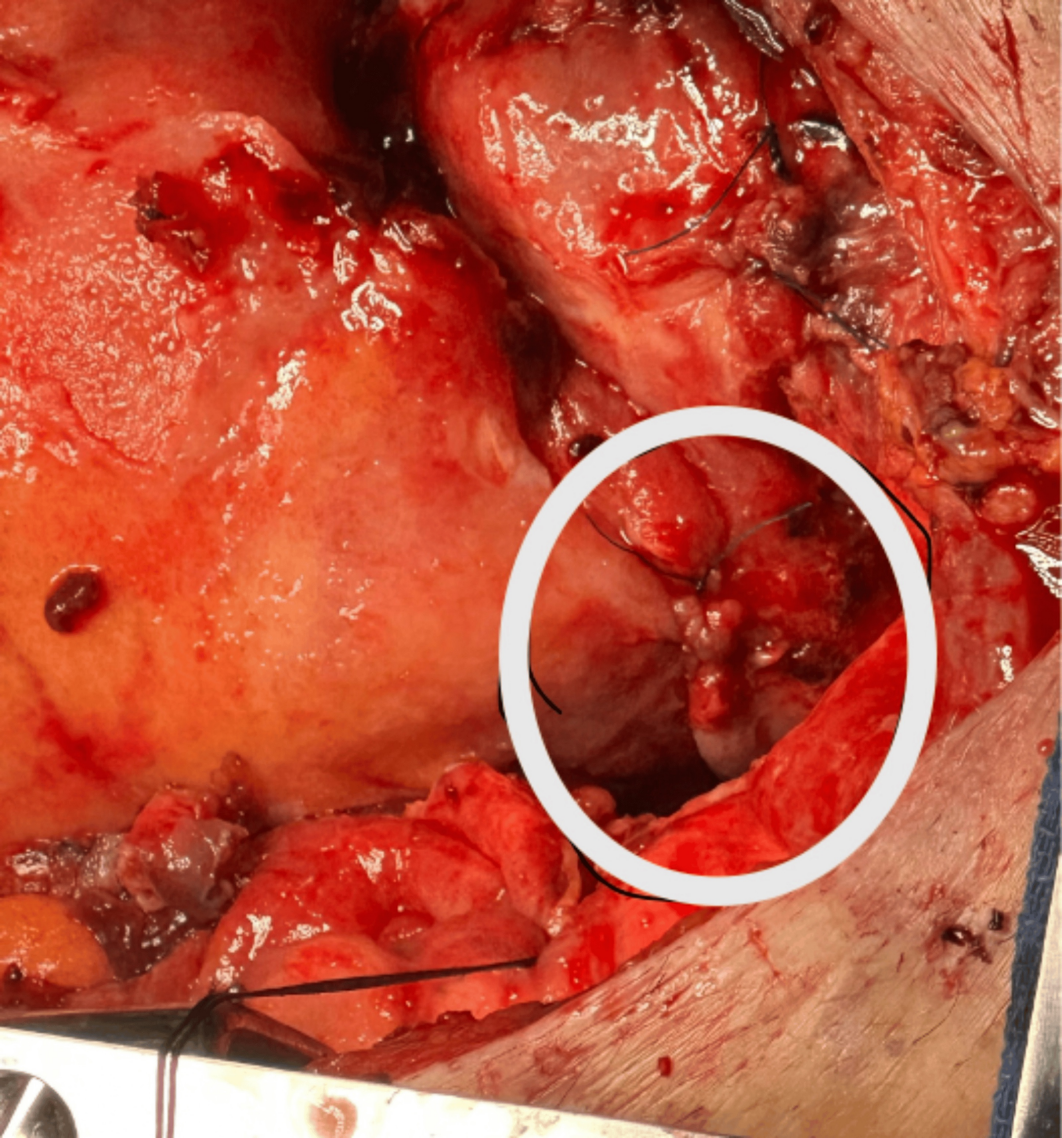
Surgical field showing the site of PAC entrapment Intraoperative view of the surgical field showing the superior vena cava (SVC) cannulation site (within the white circle) where the PAC was entrapped. PAC: pulmonary artery catheter

After median sternotomy, systemic heparinization, and ascending aortic and bicaval cannulation, cardiopulmonary bypass (CPB) was initiated. A left ventricular vent was inserted through the right superior pulmonary vein into the left atrium to facilitate ventricular decompression and optimize surgical exposure. The mitral valve (MV) was accessed via a right atriotomy, and after successful MVR, separation from CPB was successful; however, blood was observed at the PAC thermistor connector port, which reaccumulated shortly after being cleaned with an alcohol wipe, raising concern for the integrity of the PAC (Figure 1). No changes were noted in PAC pressures and waveforms. At this time, attempts to withdraw the PAC were unsuccessful. The surgical team was notified, and intraoperative assessment revealed that the PAC was entrapped at the superior vena cava (SVC) cannulation site (Figure 2). The TEE demonstrated a normally functioning bioprosthetic valve without evidence of paravalvular leak or obstruction.

**Figure 3:**
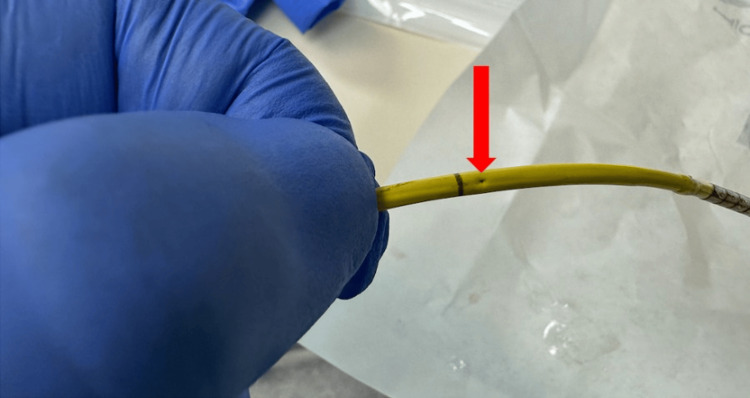
PAC post-removal PAC post-removal with needle puncture site (red arrow), likely due to surgical suture. PAC: pulmonary artery catheter

The PAC entrapment was managed surgically by releasing the SVC cannulation sutures and re-securing the cannulation site, allowing safe removal of the catheter (Figure 3). The remainder of the procedure proceeded without further complication. The patient was transferred to the cardiac surgical intensive care unit on low-dose norepinephrine infusion. Her postoperative course was unremarkable, and she was discharged from the intensive care unit to a step-down unit on postoperative day two.

## Discussion

The PAC, first described by Swan and Ganz in the 1970s as a research tool, was rapidly incorporated into clinical practice during the 1980s due to the extensive hemodynamic data and sound physiological basis it provided in critically ill patients [7]. However, its use began to decline in the 1990s following studies that indicated that this invasive technique did not confer a survival benefit. Overall PAC utilization decreased by 67.8%, with two distinct temporal patterns: a marked annual decline from 1999 to 2011, followed by stabilization through 2013 [8]. Subgroup analyses demonstrated sustained reductions in PAC use among patients with acute myocardial infarction and respiratory failure. In contrast, heart failure patients experienced an initial decline in PAC use, reaching a nadir in 2009, followed by a subsequent increase (from 9.1 PACs per 1,000 admissions in 1999 to 4.0 in 2009 and rising to 5.8 in 2013). Notably, in-hospital mortality, 30-day mortality, and length of stay decreased over the study period in this population [9]. A separate review examining patients with acute cardiogenic shock compared PAC-based versus non-PAC hemodynamic monitoring and evaluated 30-day mortality outcomes [10]. This analysis concluded that PAC use was associated with reduced in-hospital mortality in patients with cardiogenic shock, with the lowest mortality observed in those who underwent early and comprehensive hemodynamic assessment using a PAC. Thus, the PAC remains a valuable but highly selective monitoring tool in critically ill patients. American College of Cardiology and the American Heart Association (ACC/AHA) guidelines suggest considering PAC use in patients with major hemodynamic problems, such as decompensated heart failure, severe valvular disease, mixed shock states, or pulmonary hypertension that cannot be corrected before surgery [2]. Observational studies also suggest benefit in severely injured trauma patients, with benefits appearing greater in older patients, those with acute heart failure, and patients with hypotension who require inotropic support [2].

The safe and effective use of PAC depends on a careful assessment of risks versus benefits [11]. Optimal application requires proficiency in catheter insertion, accurate data collection, and skilled interpretation - competencies that may be diminishing as trainees encounter fewer opportunities for exposure. Ensuring safety relies on judicious patient selection, strict adherence to standardized insertion protocols, routine evaluation of the ongoing need for the catheter, and heightened vigilance to prevent or promptly identify complications.

PAC-related complications may be grouped into those arising during insertion (encompassing central venous access-related and the catheter insertion process-related), and those associated with the catheter’s ongoing residence. A PAC is usually inserted via a central venous introducer following induction of anesthesia and prior to surgical incision. It remains in place throughout the procedure, including during CPB, and is commonly maintained postoperatively for ongoing monitoring in the ICU. The catheter’s course through the SVC, right atrium, with looping in the right ventricle and residing in the pulmonary artery, makes it vulnerable to complications such as inadvertent fixation by surgical sutures at cannulation or vent sites, intracardiac knotting, and injury to cardiac or vascular structures. While PAC insertion was uneventful and the MVR was completed, blood was observed at the blue thermistor connector port of the PAC after weaning from CPB, as shown in Figure 1. External contamination, such as from blood sampling or surgical field spillage, was excluded, as blood reappeared despite thorough cleaning of the port. This raised concern regarding the integrity of the PAC, with the most likely source of bleeding being the thermistor lumen, suggesting catheter damage. The operating surgeon was promptly informed of the concern while a systematic evaluation of the PAC was undertaken. All ports and external connectors were inspected for abnormalities, and catheter mobility was carefully assessed. During the attempted withdrawal of the PAC, significant resistance was encountered. Concurrently, the surgeon was able to localize the point of entrapment to the SVC cannulation site, as illustrated in Figure 2. With intraoperative TEE, the PAC was visualized along its expected anatomical pathway, with no evidence of abnormal looping or migration. Evaluation of the tricuspid valve demonstrated intact leaflet structure and normal function, with no signs of entanglement, regurgitation, or valvular injury. No additional catheter-related or structural complications were identified on TEE. As the chest remained open at the time, the surgeon was able to directly address the issue by removing the implicated sutures, after which the PAC was withdrawn without difficulty. The SVC cannulation site was subsequently repaired in standard fashion. Post-removal inspection of the PAC revealed structural damage, most consistent with needle puncture, located just proximal to the thermistor heating element, as shown in Figure 3.

PAC entrapment is an under-recognized complication, particularly in the intraoperative setting. When identified postoperatively, it may necessitate re-operation and is associated with a higher morbidity as well as prolonged hospital length of stay. With a reported incidence of 0.0068%, it typically occurs intraoperatively when the surgeon inadvertently sews the PAC to a part of the heart or blood vessel during closure [5]. Other mechanisms include knotting or entanglement in the tricuspid valve apparatus or distortion of the catheter, which is more likely related to difficulties with placement or in patients with challenging anatomy, such as severe pulmonary hypertension or right heart dilation [12]. Most reported cases of PAC entrapment are associated with MV surgery, as in our case [5]. This is likely related to the multiple cannulation strategies commonly employed for the procedure, such as bicaval cannulation, atriotomy, and ventricular vent placement, which create additional opportunities for inadvertent suturing along the course of the PAC.

A distinctive feature of our case was the appearance of blood at the blue thermistor connector port of the PAC following weaning from CPB and removal of the cannulas. Continuous cardiac output PACs include a thermistor located 4 cm from the tip and a thermal filament port connected to a heating element located approximately 14 to 25 cm from the distal tip, which enables cardiac output measurement via thermodilution. Compromise of PAC integrity, due to entrapment by a surgical suture as in this case, likely allowed blood to track to the thermal filament connector port. Other clues to an entrapped PAC include inaccurate or a significant change in values, like cardiac output noted after PAC manipulation in the surgical field, inability to move or unusual resistance during attempted repositioning, hemodynamic instability following withdrawal after resistance is felt indicative of possible pulmonary artery rupture that must be immediately addressed [13], and echocardiographic clues such as close approximation of the PAC to an echogenic area consistent with suture material or tenting of the involved structure during traction [14].

Therefore, we recommend routine confirmation of PAC mobility during all cardiac surgical procedures before chest closure. This may be accomplished by withdrawing the catheter approximately 5 to 10 cm while the surgeon directly observes for retraction at sites where cannulation or suturing has occurred along the PAC’s course [5]. We emphasize that withdrawing at least 5-10 cm is necessary, as smaller movements may merely straighten a looped catheter, falsely suggesting the absence of retraction in the surgical field or resistance to withdrawal. Although this maneuver does not prevent entrapment, it facilitates early recognition and timely surgical intervention. However, it does not exclude the presence of catheter coiling or knotting. TEE may further aid in prevention by allowing visualization of the catheter tip movement within the main pulmonary artery during withdrawal [14]. PAC entrapment, if not detected intraoperatively, is associated with increased morbidity, longer hospital stays, and delayed recovery.

In practice, PAC entrapment is most often discovered in the postoperative period when attempting to reposition the catheter or at the time of removal. Diagnosis relies on a high index of suspicion when resistance is encountered during catheter removal. One must avoid forceful removal and initiate a prompt imaging assessment. TEE and fluoroscopy are valuable tools for identifying the site and mechanism of entrapment. It can lead to major bleeding, cardiac tamponade, or cardiac rupture, especially if forceful removal causes papillary muscle rupture. PAC entrapment can be associated with life-threatening complications such as cardiac rupture, pulmonary artery rupture, tamponade, severe tricuspid regurgitation, or arrhythmias. Management is typically a re-operation where the catheter is freed by releasing the sutures, and then can be easily removed. Definitive treatment most often requires redo sternotomy for surgical retrieval (in 75% of cases), though percutaneous techniques may be used for knotting or snaring complications [5,12].

## Conclusions

An entrapped PAC is commonly detected postoperatively when the catheter is being discontinued. In this case, the presence of blood at the thermistor port served as an important clue, prompting recognition of the complication and notification of the surgeon. This allowed intraoperative confirmation of PAC entrapment and timely management while the chest remained open. Our recommended strategy is to routinely check for PAC mobility prior to chest closure, as visualization of blood at the thermistor port is not a common presentation. This case underscores the importance of a multidisciplinary approach, with prevention relying on meticulous intraoperative handling and heightened vigilance for catheter entrapment before chest closure.
